# The Impact of Individual Mobility on Long-Term Exposure to Ambient PM_2.5_: Assessing Effect Modification by Travel Patterns and Spatial Variability of PM_2.5_

**DOI:** 10.3390/ijerph18042194

**Published:** 2021-02-23

**Authors:** Eun-hye Yoo, Qiang Pu, Youngseob Eum, Xiangyu Jiang

**Affiliations:** 1 Department of Geography, State University of New York at Buffalo, Buffalo, NY 14260, USA; qiangpu@buffalo.edu (Q.P.); yeum@buffalo.edu (Y.E.); 2 Georgia Environmental Protection Division, Atlanta, GA 30354, USA; xiangyuj@buffalo.edu

**Keywords:** long-term exposure to ambient PM_2.5_, uncertainty, mobility-based approach, spatial exposure models, routine travel patterns

## Abstract

The impact of individuals’ mobility on the degree of error in estimates of exposure to ambient PM${}_{2.5}$ concentrations is increasingly reported in the literature. However, the degree to which accounting for mobility reduces error likely varies as a function of two related factors—individuals’ routine travel patterns and the local variations of air pollution fields. We investigated whether individuals’ routine travel patterns moderate the impact of mobility on individual long-term exposure assessment. Here, we have used real-world time–activity data collected from 2013 participants in Erie/Niagara counties, New York, USA, matched with daily PM${}_{2.5}$ predictions obtained from two spatial exposure models. We further examined the role of the spatiotemporal representation of ambient PM${}_{2.5}$ as a second moderator in the relationship between an individual’s mobility and the exposure measurement error using a random effect model. We found that the effect of mobility on the long-term exposure estimates was significant, but that this effect was modified by individuals’ routine travel patterns. Further, this effect modification was pronounced when the local variations of ambient PM${}_{2.5}$ concentrations were captured from multiple sources of air pollution data (‘a multi-sourced exposure model’). In contrast, the mobility effect and its modification were not detected when ambient PM${}_{2.5}$ concentration was estimated solely from sparse monitoring data (‘a single-sourced exposure model’). This study showed that there was a significant association between individuals’ mobility and the long-term exposure measurement error. However, the effect could be modified by individuals’ routine travel patterns and the error-prone representation of spatiotemporal variability of PM${}_{2.5}$.

## 1. Introduction

Environmental health studies have routinely conducted exposure assessments using pollutant measurements from sparse networks of monitors matched with the locations of home addresses of individuals [1,2]. However, reliance on these networks leads to measurement error in exposure assessments because they are unable to capture high spatial heterogeneity in pollution fields [3,4]. Similarly, this approach, which focuses only on individuals’ location of residences, ignores time-activities of individuals and fails to assess dynamic exposure to air pollution [5,6,7,8,9]. In sum, considerable uncertainties exist in residence-based exposure assessments relying on air monitoring networks.

As an effort to improve the resolution and availability of air pollution data, recent studies have incorporated additional data sources, such as satellite images and computer simulation outcomes, to supplement monitoring measurements. Particularly, satellite-derived aerosol optical depth (AOD) and the Community Multiscale Air Quality Modeling System (CMAQ) have received growing interests as they provide fine spatiotemporal data with complete spatial coverage. It is worth noting, however, that the estimation of spatial and temporal variability of ambient air pollutant concentration alone cannot guarantee the accuracy and precision of personal exposure estimates [10,11,12,13,14,15].

Similarly, several epidemiological studies attempted to reduce exposure misclassification by accounting for the variations in the time people spend in different locations by using time-weighted pollutant concentrations in each microenvironment [16,17,18,19]. These studies typically collected participants’ activity data and their locations using 24-h diaries in a survey [20], which involves high costs and raises concerns regarding reliability and validity [21,22]. Recent technological advancements, such as global positioning system (GPS) and mobile phones, and their adoption in exposure assessments have shifted the paradigm in exposure studies [23,24,25,26]. Unlike traditional travel surveys, mobile phones equipped with GPS provide detailed and accurate time–activity information with low burden to the phone carriers and low cost to researchers [27,28]. These mobile phones are also able to frequently measure and record their locations. Researchers can use the resulting geographic coordinates (latitude and longitude) to measure an individual’s locations at multiple points throughout ones’ daily life instead of relying solely on a person’s residential location to estimate his or her air pollutant exposures [2,29,30].

Despite a continuous effort to understand the relationship between ambient air pollution and personal exposures, previous investigations of exposure measurement error and exposure misclassification have been confined to a single issue. Some studies have focused on the mischaracterization of the spatial heterogeneity of air pollution due to the limited availability and resolution of environmental pollution data [31,32,33,34,35]. Other studies investigated the effects of ignoring human mobility on air pollution exposures [36,37,38,39,40,41]. The uncertainty of personal exposure to ambient air pollution is likely affected by both the representation of spatiotemporal variability of air pollution and the use of time–activity data to capture dynamic nature of individuals’ movements [28,42,43,44]. However, few studies systematically investigated the joint effect of the human mobility and spatial exposure models on the uncertainty of the personal exposure assessment.

This gap is important to fill because exposure measurement error is likely driven by a synergy of two related factors. Assessment based on location-specific exposure at the home address rather than mobility-based exposure contributes to exposure measurement error. However, the degree of accuracy versus error in these home-based estimates will likely vary based on individuals’ routine travel patterns and/or how the spatiotemporal dynamics of air pollutant concentrations are modeled. For example, the exposure measurement error induced by failing to account for daily mobility will be much smaller for individuals who typically spend most time at home compared to people who regularly travel long distance and spend much time outside the home [45]. Similarly, personal exposure with and without taking one’s daily mobility into account would make little to no difference if a spatial exposure model fails to accurately represent the spatiotemporal variability of air pollution.

An increasing number of studies have demonstrated the effect of mobility on individuals’ exposure to air pollution, although most studies are based on either simulated or approximated mobility data, such as call detail records. Some studies have conducted using empirical data or accurate mobility data, but they often lack generalizability due to a small sample size and/or a short study term. For example, Dons et al. [46] assessed personal exposure to black carbon using real time location data collected from GPS loggers of 16 participants over 7 days, whereas Park and Kwan [38] compared individuals’ exposure to O${}_{3}$ with and without consideration of 80 participant’s simulated activities on a single day. Other studies were conducted with a large number of participants, but individuals’ location data are approximated by nearest cell tower locations [2,30,47]. This discrepancy between actual and approximated movement data may lead to exposure measurement error in longitudinal studies of air pollution and health, particularly if they were matched with spatially and temporally resolved air pollutant estimates. Lastly, most studies that accounted for individuals’ mobility tend to focus on short-term exposure (from a few minutes to 24 h at best) to air pollution unless simulated data are used [48,49,50]. It is clear that the effect of mobility on longer term exposure over months, referred to as long-term (or sub-chronic) exposure hereafter, using empirical and accurate time–activity data with sufficiently large participants has been understudied.

In the present study, we examined the effect of individuals’ mobility on long-term air pollution exposure measurement error using accurate and real-world time–activity data collected from thousands of participants in the study area over a few months. We also investigated individuals’ routine travel patterns and spatial exposure models as moderators of the effect of an individual’s mobility. First, we examine whether individuals’ routine travel patterns moderate the impact of mobility on individual long-term exposure assessment. Next, we examine the role of the spatiotemporal representation of ambient fine particulate matter (PM${}_{2.5}$) concentrations as a second moderator of the relationship between individuals’ mobility and the exposure measurement error. We predict a three-way interaction effect of spatial exposure models, routine travel patterns, and individuals’ mobility on long-term exposure measurement error to ambient PM${}_{2.5}$ concentrations using the data collected in Erie and Niagara counties, New York, NY, USA.

## 2. Materials and Methods

### 2.1. Time–Activity Data

We used time–activity information of 2013 residents in the Erie and Niagara counties, New York State (NYS), from 1 December 2016 to 31 May 2017. Most participants began their survey in October or November of 2016, and participated in the study for a minimum of 72 days to 182 days. To ensure the continuous monitoring, we retained the mobile phone traces observed between December 2016 and May 2017. At the beginning of the study we collected the address of residence and workplace (if applicable) of participants. Individuals’ whereabouts during the study period were collected using their own mobile phone and an application developed for Android and iOS systems by our research team. Specifically, mobile phone traces were periodically reported by the Android application every 35 min on average with a high accuracy (40 m of positional error). For iPhone users, their movement information was updated whenever the device moved more than 500 m from the last recorded location. The iOS system-based location data had a high positional uncertainty of 650 m, but they had systematic temporal gaps. We filled these gaps using a set of imputation strategies with a 30-min imputation interval, while taking into account self-reported place information provided by study participants and positional uncertainty of the data. Detailed information on the data and the imputation strategies are available in Yoo et al. [51].

Still, we found that substantial amounts of daily time–activity information were missing to calculate individuals’ daily time-integrated exposure to ambient PM${}_{2.5}$. To address this issue, we developed a deep long short-term memory imputation model to predict the numerical representation of time–activity for the days. Here, we replaced the missing time–activity with the daily observations which are the most similar to the predicted numerical representation. We imputed missing time–activity data at every 30 min-interval and assigned the place at which the activity took place. We evaluated the performance of imputation in terms of the accuracy of reproducing time–activity patterns of individuals, such as daily average time spent at home, work, and shopping places each day.

### 2.2. Air Pollution Data

We considered two spatial exposure model scenarios in this study to reconstruct spatial variability of daily ambient PM${}_{2.5}$ concentrations in 2016. First, we generated estimates of daily PM${}_{2.5}$ concentrations using a single-sourced data, that is, measurements obtained from the monitoring networks [7]. There was a limited number of monitoring stations in the range of 1 to 5 stations operated in Eire/Niagara county during the study period of 2016. To improve the reliability of predictions, we used a total of 38 monitoring stations present in NYS for model building. Monitoring data used in the present study were obtained from the U.S. Environmental Protection Agency (EPA, http://www.epa.gov/).

In the multi-sourced exposure model, we compensated the lack of spatial coverage of monitoring station data by incorporating both satellite-derived Aerosol Optical Depth (AOD) and the Community Multi-scale Air Quality Model (CMAQ) outputs. Satellite-derived AOD does not directly represent ground-level PM${}_{2.5}$ concentrations, and thus they need to be calibrated by accounting for the influences from meteorological, topological and anthropogenic conditions [52,53]. In the present paper, we reconstructed a daily PM${}_{2.5}$ concentration surface over the study area primarily based on the 1 km high resolution AOD derived by the algorithm of Multi-Angle Implementation of Atmospheric Correction (MAIAC) [54,55,56]. During the study period, however, there were many days without sufficient MAIAC AOD retrievals due to cloud/snow presence. To address this missing AOD problem, we imputed the missing values by incorporating multiple proxy data available at coarse spatial resolutions, including Modern-Era Retrospective Analysis for Research and Applications version 2 and the CMAQ model outputs [57].

### 2.3. Measures

#### 2.3.1. Individual’s Travel Patterns

We examined daily travel patterns of each participant using spatial and temporal metrics, namely, the characteristic distance travelled by individuals and the time allocated at places other than one’s home address [58,59,60]. Specifically, we calculated a radius of gyration (RoG), denoted by $r_{g}^{j}$, on a daily basis as

(1) $$r_{g}^{j}=\sqrt{\frac{1}{N_{j}}\sum_{k\in Lj}n_{k}{\left(u_{k}-u_{c_{j}}\right)}^{2}},$$

to characterize the distance travelled by a participant on a day *j*. Here, $L_{j}$ denotes the set of locations visited by a participant on the day *j*; $u_{k}$ is a two-dimensional vector of geographic coordinates of location *k*; $n_{k}$ is the frequency of visits at the *k*-th location; $N_{j}=\sum_{k\in L_{j}}n_{k}$ is the total number of trips made on the day *j*; and $u_{c_{j}}$ is the center of mass of the individual on the day *j*. The daily characteristic distance of *i*-th participant (i=1,…,2013) was further aggregated as an average RoG $r_{i}=\frac{1}{n_{i}}\sum_{j=1}^{n_{i}}r_{g}^{j}$ over the number of days $n_{i}$ that the participant *i* enrolled in the survey. We also calculated each participant’s daily averaged time spent away from home (referred to as non-home time), denoted as $\tau_{i}$, over the study period. Considering the positional error of mobile phone location data, we used a buffer of 2 km centered at each participant home address and excluded all GPS trajectories within the buffered zones in the calculation of $\tau_{i}$.

Based on these two metrics, we classified study participants into three groups: (1) individuals with static travel patterns who spend most time at home; (2) individuals with moderate travel patterns as they either travel long-distance but spend little time outside the home or travel short distances but spend many hours outside the home; (3) individuals with active travel patterns who regularly travel long-distance and spend large amount of time away from home. The classification scheme is summarized in Table 1, where we used the mean distance ($R_{c}$) of daily RoGs in pooled study participants and the mean non-home time ($\Gamma_{c}$) of the study participants during the study period, respectively.

#### 2.3.2. Spatial Variability of Daily PM${}_{2.5}$ Concentration

We used the two spatial exposure models to reconstruct the daily PM${}_{2.5}$ variations across the study area. In the single-source exposure model, we estimated daily PM${}_{2.5}$ concentrations throughout the study area using Ordinary Kriging (OK), known as a best linear unbiased estimator among spatial interpolation methods [61,62,63]. In the multi-sourced exposure model, we used an ensemble model that integrates multiple machine-learning algorithms based on its superior performance to a single machine learning algorithm [64,65].

The two exposure models differ substantially in terms of the data sources that they relied on rather than their statistical approaches. Both spatial interpolation techniques make predictions across space from air monitoring data and rely on relationships being identified within the data itself or with information regarding the surrounding environment [66,67]. Both models generate predictions of daily PM${}_{2.5}$ concentration across the entire study area using some forms of spatial dependence, which are quantified as a mathematical function of the distance between monitoring sites. When sparsely sampled air monitoring data are used as the primary source in the single-sourced exposure model, an overly-smooth spatial structure of daily PM${}_{2.5}$ concentrations is produced with high levels of spatial uncertainty. Meanwhile, the multi-sourced model captures local variations of PM${}_{2.5}$ concentrations by incorporating additional information from AOD and auxiliary variables, such as land cover, population density, and meteorological conditions.

The performance of the two exposure models was evaluated using the 10-fold spatial cross validation (CV) method. Specifically, the monitoring sites within NYS in 2016 were randomly divided into 10 groups where sites from nine groups were used as training sites and the remaining sites were used for validation. We trained each model with the same training data aggregated from the training sites and made predictions at validation sites. The differences between predicted and known values at validation sites were summarized by metrics, such as root mean square error (RMSE) and mean prediction error (MPE). This process was repeated 10 times until all monitoring sites were selected as validation sites.

#### 2.3.3. Personal Exposures to PM${}_{2.5}$ Concentrations

We estimated personal exposure to ambient PM${}_{2.5}$ using both a residence-based and mobility-based approach. Specifically, we matched home address and mobile-phone traces with a daily PM${}_{2.5}$ concentration surface estimated from the two spatial exposure models (single-sourced and multi-sourced models), respectively. It should be noted that PM${}_{2.5}$ monitoring data and satellite-derived AOD are obtained for the entire year of 2016, but the time–activity data are collected across the years of 2016 and 2017. Here, the primary focus lies on the measurement error by ignoring participants’ whereabouts instead of the actual exposure estimates and thus we treated the time–activity data collected in 2017 (January to May) as observations in 2016 and matched them with corresponding PM${}_{2.5}$ estimate surfaces.

For each participant we estimated four sets of daily exposure to PM${}_{2.5}$ and aggregated them over time for long-term exposure. Let $y_{M}^{S}\left(i,j\right)$ be the PM${}_{2.5}$ exposure estimate of participant i=1,…,2013 on day $j=1,\ldots ,n_{i}$ from a PM${}_{2.5}$ surface estimated from the *S* exposure model. Here, $n_{i}$ denotes the total number of days that the *i*-th subject participated the study. Each day we also estimated a pair of daily PM${}_{2.5}$ exposures with (M=1) and without (M=0) one’s time–activity patterns. The residence-based exposure was calculated by extracting the PM${}_{2.5}$ concentration estimates at each participant’s home address, where daily PM${}_{2.5}$ surfaces were obtained from a single-sourced (S=0) and a multi-sourced (S=1) exposure model, respectively. For the mobility-based exposure, we used the general form of the equation where the time-weighted exposure was calculated as [68]

(2) $$y_{M=1}^{S}\left(i,j\right)=\sum_{k\in L_{j}}C_{k}^{S}t_{k},$$

where $C_{k}^{S}$ and $t_{k}$ are the PM${}_{2.5}$ concentration estimated from the *S*-source exposure model (S=0,1) and the total time spent at the *k*-th place located at $u_{k}$ on the day *j*, respectively. The daily exposure to ambient PM${}_{2.5}$ concentrations of each study participant was further summarized into a long-term exposure by taking the average of the *i*-th participant’s daily exposure estimates as $Y_{M}^{S}\left(i\right)=\frac{1}{n_{i}}\sum_{j=1}^{n_{i}}y_{M}^{S}\left(i,j\right)$ for S=0,1 and M=0,1.

### 2.4. Statistical Analyses

We developed a 3-way interaction model in a linear mixed-effect regression to study the effect of the mobility on exposures to ambient PM${}_{2.5}$ concentrations. Here, we also explicitly accounted for the moderation by other two factors—individuals’ routine travel patterns and spatial exposure models. Three sets of analyses were conducted to address each hypothesis below:

**Hypothesis** **1.** *The consideration of individuals’ mobility affects long-term exposure to ambient PM* ${}_{2.5}$ *concentrations.*

**Hypothesis** **2.** *Routine travel patterns moderate the relationship between the mobility and estimates of the individuals’ exposure to ambient PM* ${}_{2.5}$ *concentrations.*

**Hypothesis** **3.** *The type of spatial exposure models (multi-sourced or single-sourced) moderates the two-way interaction between of mobility and the long-term travel patterns on estimates of individual exposure to ambient PM* ${}_{2.5}$ *.*

To test each hypothesis, we developed a multiple regression model with additional interaction terms to determine whether a moderating effect exists. We also accounted for the potential correlation among multiple exposure measures per participant by developing a random effect model.

In the statistical analyses, we used an indicator coding scheme for the mobility effect. It is worth noting that our primary focus was on the statistical significance of the relative difference of mobility-based exposures from residence-based exposures. We anticipated that the sign or the magnitude of the differences (i.e., exposure measurement errors) might vary for pollutants other than PM${}_{2.5}$ or different study regions. However, the statistical significance of the differences implies that the consideration of individuals’ mobility affects the exposure measurement error. For individuals’ routine travel patterns, we used a numerical variable (referred to as ‘activity-travel score’), which coded static, moderate and active travel patterns as a numerical value of 0, 1, and 2, respectively. This new coding scheme facilitates the interpretation of the statistical analysis. For example, we can relate the changes in the long-term exposure to ambient PM${}_{2.5}$ as a function of individuals’ travel pattern in terms of the travel distance and non-home time. This coding also enabled us to account fo the larger differences between static and active travel patterns versus moderate and active travel patterns.

The statistical analyses, spatial prediction of PM${}_{2.5}$ concentrations and time–activity pattern analyses were conducted in R version 4.0.2 (R Core Team, 2020). We used the nlme package (v.3.1-148) and the effects package (4.2-0) for statistical analyses and visualizations; and the gstat package (v.2.0.6) for a single-sourced exposure model. We used a fast and scalable machine learning platform, H2O (https://www.h2o.ai/) (accessed on 3 February 2021), to build the multi-sourced ensemble model.

## 3. Results

### 3.1. Daily Mobility Patterns and Routine Travel Patterns

A total of 2013 participants’ time–activity data collected between 1 December 2016 and 31 May 2017 were included in the study. For each participant we reconstructed time–activity patterns at 30-min intervals each day during the study period. During the six months of the study period, a total of 1612 to 2012 participants engaged in the survey each day. Based on the reconstructed time–activity trajectories, we characterized each participant’s routine travel patterns by calculating both the averaged radius of gyration ($r_{i}$ in km) and the averaged time spent away from home ($\tau_{i}$ in hour). The averaged daily travel distance measured in the averaged radius of gyration (RoG) and the time spent away from home (NhT) of each study participant are summarized in Table 2. The pooled and average travel distance, characterized by $R_{c}=$ 6.27 km with a standard deviation 3.60 km with a range of 0.08 to 24.34 km. The study participants also spent their time away from home on average $\Gamma_{c}=$ 5.84 h, with a standard deviation of 2.91 h.

**Figure 1:**
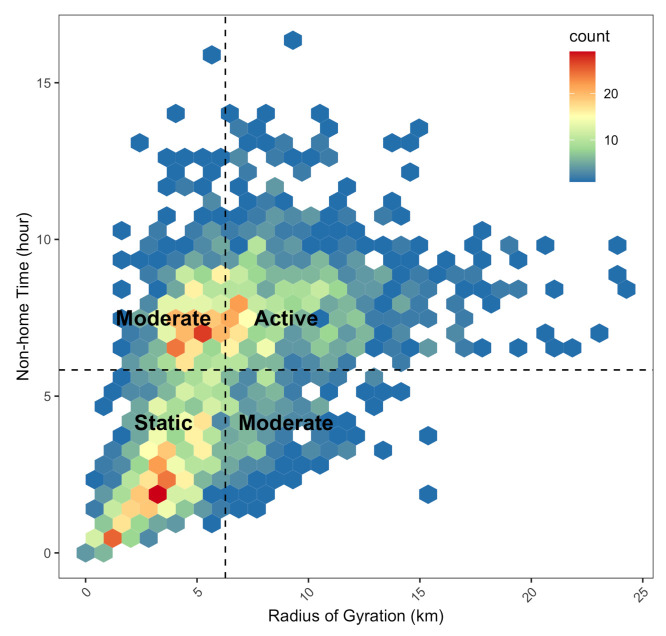
Stratification of individuals’ travel patterns based on the averaged travel distance (RoG) of 6.27 km and time spent away from home (Non-home time) of 5.84 h.

Based on the averaged daily travel distance and time spent outside the home, we classified study participants into three groups. Study participants whose averaged RoG and NhT are greater than 6.27 km and 5.84 h were considered as active travelers (Active in Figure 1). In contrast, if their RoG and NhT are both less than 6.27 km and 5.84 h, we classified their mobility patterns into static (Static in Figure 1). The rest of the study participants were considered to have moderate travel patterns. The classification scheme is illustrated in Figure 1. The total number of participants for Static, Moderate, and Active group is 720 (35.8 %), 654 (32.0%), and 648 (32.2%), respectively.

### 3.2. Spatial and Temporal Variability of PM${}_{2.5}$ Concentrations

Over the study period of six months (January to May, and December) in 2016, the spatial average of daily PM${}_{2.5}$ concentration in the study region varied from 1.25 µg/m${}^{3}$ and 16.58 µg/m${}^{3}$ with a mean of 7.15 µg/m${}^{3}$ and standard deviation of 3.11 µg/m${}^{3}$ from the single-sourced exposure model and a mean of 5.60 µg/m${}^{3}$ and standard deviation of 2.28 µg/m${}^{3}$ from the multi-sourced exposure model. The daily mean and standard deviation of PM${}_{2.5}$ estimates obtained from two models were calculated over the six months and we summarized their distributions in Table 3. Overall, the average of daily PM${}_{2.5}$ concentration estimated from the multi-sourced exposure model was lower than the averaged PM${}_{2.5}$ predictions obtained from the single-sourced model. However, the multi-sourced model captured higher levels of spatial variability (1.16 of the multi-sourced model versus 0.25 of the single-sourced model). The standard deviation (SD) of daily PM${}_{2.5}$ concentration estimates, which quantifies how daily PM${}_{2.5}$ predictions vary in space, was also substantially different between the two models. The multi-sourced exposure model predictions have higher variability with a mean of 1.16 than a single-sourced model estimates whose mean SD was as low as 0.25.

**Figure 2:**
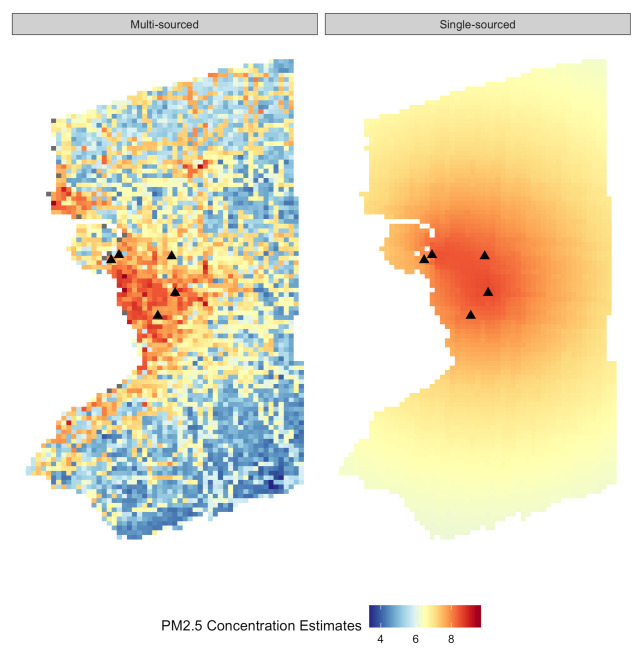
PM${}_{2.5}$ estimates obtained from both a multi-sourced and single-sourced exposure models on 1 December 2016 overlaid with the five air monitoring stations operated in 2016 (denoted as symbols of black triangle).

As illustrated in Figure 2, the PM${}_{2.5}$ predictions of a single day (12/01/2016) obtained from the single-sourced exposure model in the right panel resulted in smoother spatial structures than the multi-sourced exposure model in the left panel. This is highly anticipated because the smoothing effect of the Ordinary Kriging is well known by geostatisticians and is considered as a serious drawback [69]. Specifically, the smooth patterns of PM${}_{2.5}$ prediction in the right panel of Figure 2 is a result of making predictions from sparsely sampled air monitoring data (denoted as the symbol of triangles), where low levels of PM${}_{2.5}$ predictions were overestimated and high values were underestimated. Meanwhile, the PM${}_{2.5}$ prediction from the multi-sourced model in the left panel of Figure 2 depicted local variations of daily PM${}_{2.5}$ concentrations that were affected by the proximity to road networks, the spatial variations of population density and land use type. These visual inspections were also asserted by the spatial 10-fold cross validation (CV), where the multi-sourced model outperformed the single-sourced model with CV-R${}^{2}$ of 0.74 versus 0.66. A similar pattern was found in the comparison of RMSE/MPE of the two models: 1.91/1.40 µg/m${}^{3}$ for the multi-sourced model and 1.96/1.49 µg/m${}^{3}$ for the single-sourced model.

**Figure 3:**
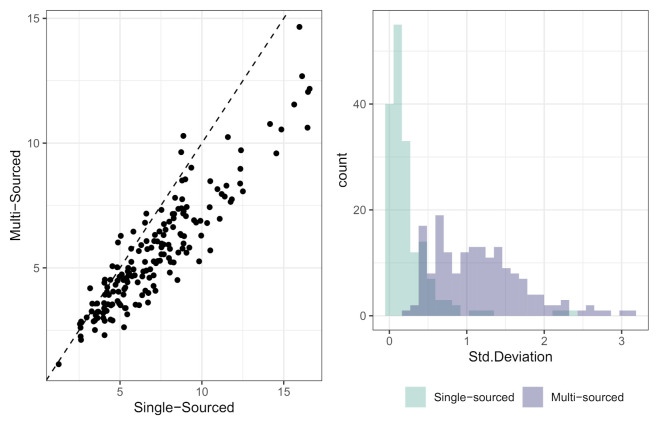
PM${}_{2.5}$ concentration estimates obtained from both a multi-sourced and single-sourced exposure models and the comparison of model predictions using the daily spatial mean and standard deviations.

Lastly, we compared the spatial averaged PM${}_{2.5}$ concentration levels from both models at each day during the study period. This comparison is significant because the daily spatial variation of PM${}_{2.5}$ predictions could directly affect personal exposure estimates. As presented in Figure 3, the single-sourced model consistently yielded higher PM${}_{2.5}$ concentrations than the multi-sourced model except a few days (see the left panel). We also examined the distribution of spatial variation of the estimated daily PM${}_{2.5}$ concentrations from both models using the histogram in the right panel. The standard deviation of the estimates across the study area is much smaller for a single-sourced model compared to the multi-sourced model, suggesting that the single-sourced model might result in over-smoothed PM${}_{2.5}$ predictions.

### 3.3. Mobility Effect on Long-Term Exposure to Ambient PM${}_{2.5}$

For each participant, we calculated four long-term exposure estimates obtained from two spatial exposure models of ambient PM${}_{2.5}$ with and without the individual’s mobility. Based on the four exposure estimates of 2013 participants, we investigated the effect of personal mobility on individual exposure to ambient PM${}_{2.5}$. Considering that the contribution of mobility in personal exposure assessment may vary per individuals’ travel patterns and the spatiotemporal variability of air pollution fields, we formally tested their interactions in the random-effect regression model. We separately assessed their interactions per a spatial exposure model that captures different levels of spatial variability of PM${}_{2.5}$ concentrations.

Specifically, we conducted multiple regression analyses to evaluate whether the consideration of mobility patterns affects the air pollution exposure assessment (i.e., measurement error). The results of analyses are summarized in Table 4. As shown in Step 1, Hypothesis 1 predicted that mobility-based exposure has a significant effect on individual exposure to ambient PM${}_{2.5}$ (β=0.03,p<0.01).

Hypothesis 2 predicted the effect of mobility on personal exposure measurement error would vary depending on individuals’ routine travel patterns. For example, mobility-based exposure would be higher for individuals with active travel patterns than individuals who travel long distances but do not spend much time outside the home or who travel short distances although spend much time outside the home (moderate travel patterns). As shown in Table 4 (Step 2), the interaction effect of mobility and individuals’ travel patterns were statistically significant (β=0.03,p<0.05).

Hypothesis 3 predicted that the effect of mobility on personal exposure measurement error would be substantial when the PM${}_{2.5}$ concentration is estimated from a multi-sourced exposure model and for individuals whose travel patterns are active—routinely travel long distances and spent a lot of time outside the home. As shown in the Step 3 of Table 4, the three-way interaction effect of mobility, active travel patterns of individuals, and a multi-sourced exposure model on personal exposure to PM${}_{2.5}$ was significant (β=0.05,p<0.05).

**Figure 4:**
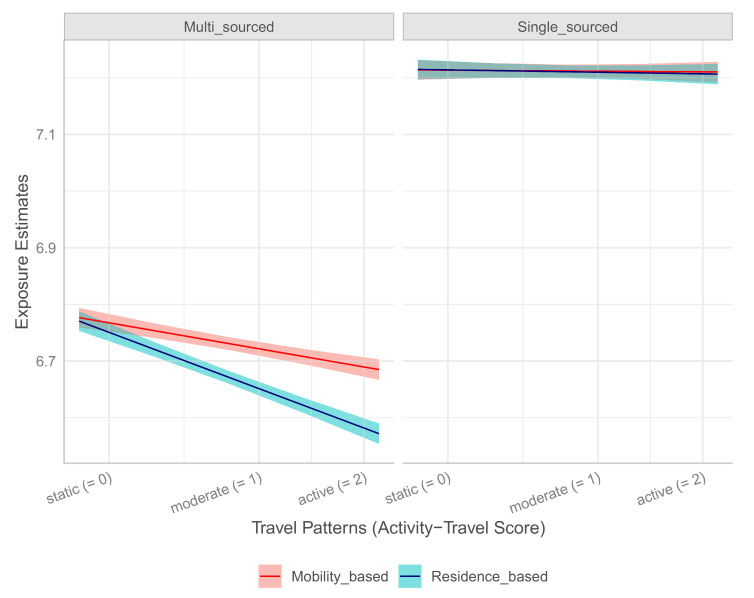
The three-way interaction effects of individuals’ daily mobility, routine travel patterns, and the exposure models on long-term exposure to ambient PM${}_{2.5}$ concentrations.

To facilitate the understanding of this significant three-way interaction, we plotted the effect of mobility patterns on personal exposure in Figure 4. The long-term exposure estimates from the single-sourced model are substantially higher than the multi-sourced model. Further, there was no effect of travel pattern on exposure estimates when the single-sourced model was used regardless of whether they were based mobility or residence (in the right panel). In contrast, when the daily ambient PM${}_{2.5}$ was predicted from the multi-sourced exposure model, the effect of mobility varied depending on whether an individual’s travel pattern is static or active (in the left panel). Greater activity-travel scores were associated with lower levels of exposure estimates, when individuals’ long-term exposure was estimated at residence. That is, the time-weighted exposures taking into account both multiple places visited by individuals and the time spent at each place was higher than one’s residence-based exposure.

## 4. Discussion

To better understand the effect of mobility on individuals’ long-term exposure to ambient PM${}_{2.5}$, we developed a statistical model for mobility, individuals’ routine travel patterns and spatial exposure models. The results strongly suggested that mobility has a statistically significant effect on personal long-term exposure to ambient PM${}_{2.5}$. Our unique contribution to existing literature, however, lies in that we demonstrated that the mobility effect can be modified by individuals’ routine travel patterns and the validity of spatial exposure models. For individuals with static travel patterns over an overly smooth PM${}_{2.5}$ surface, we found that the mobility-based approach has no effect on the accuracy of long-term exposure estimates. On the other hand, the mobility-based approach will be the most effective to capture accurate exposure of individuals with highly active travel patterns over an air pollution field with high spatial and temporal variability.

In the first stage, we examined the effect of individuals’ mobility on long-term exposure to ambient PM${}_{2.5}$ by comparing a location-specific exposure at home with time-weighted exposure based on mobility data. The results suggested that the mobility effect on individuals’ long-term exposure to ambient PM${}_{2.5}$ was statistically significant. When mobility was taken into account, personal exposure to PM${}_{2.5}$ was 0.03 µg/m${}^{3}$ higher than overall mean exposure. Our finding on the significant effect of mobility on long-term exposure is in line with previous studies [40,41,50]. To our best knowledge, however, this is one of a few studies to examine and show the statistically significant effects of the mobility on long-term exposure to air pollution in a large sample over a period of multiple months.

In the second stage, we examined effect modification by individuals’ routine travel patterns by introducing the two-way interaction term between the mobility and individuals’ travel patterns in the multiple regression model. The results revealed that the single-sourced exposure model contributed to higher long-term exposure estimates to ambient PM${}_{2.5}$ than the multi-sourced exposure model. Based on the cross-validation results, we speculated that the higher values of exposure estimates were attributed to the overestimation of PM${}_{2.5}$ predictions from the single-sourced exposure model. This overestimation from the single-sourced model is rather anticipated because the primary source of PM${}_{2.5}$ measurements obtained from air quality monitoring sites operated in the study region in 2016 are clustered around highly polluted areas, including Tonawanda Coke Facility [70].

Another unique point of our study is that we have used complete and relatively accurate mobility data collected from thousands individuals during their daily life. As demonstrated in our comparison study, however, the effect of mobility on the measurement error of long-term air pollution exposure estimates is only pronounced when they are matched with spatially and temporally resolved PM_2.5_ estimates [57]. The difference between residence-based and mobility-based exposure estimates have not been detected unless the spatial dynamics of ambient PM${}_{2.5}$ were captured by the multi-sourced model. This indicates the measurement error of long-term exposure can be reduced only by improving the spatial and temporal resolution of both mobility data and the representation of air pollution fields simultaneously.

It is important to note that our conclusions only pertain to the uncertainties associated with personal long-term exposure to ambient PM${}_{2.5}$. In contrast, our finding cannot speak to the issue of the accuracy of exposure assessment. Given that the true exposure of individuals is unknown, the accuracy (or error) of exposure assessment can not be quantified [71]. However, we attempted to quantify and compare uncertainties in long-term PM${}_{2.5}$ exposure estimates that arise from the oversimplified assumption (‘residence-based approach’) on individuals’ spatial behaviors. We further examined how the uncertainties associated with individuals’ mobility effect on one’s long-term exposure are modified by individuals’ routine spatial behaviors and the limited representation of spatiotemporal representation of air pollution fields [72].

The main strengths of this study are as follows—first, we examined the effect of mobility on individuals’ long-term exposure to ambient PM${}_{2.5}$ and highlighted the moderating effects of their routine travel patterns and the spatiotemporal dynamic of air pollution fields. Second, we conducted the study using both accurate and spatially and temporally resolved time–activity data from thousands of participants over several months, as well as high-resolution satellite-derived AODs and CMAQ model outputs to improve the availability and resolution of air pollution data [31]. As a result, the effects of small sample size and sample bias on the accuracy of personal exposure assessment were reduced. Third, we found that individuals’ mobility has a significant effect on long-term exposure to ambient PM${}_{2.5}$ in this study, although its effect was moderated by individuals’ routine travel patterns and the spatial variability in the study area. Our findings may provide a scientific basis for long-term exposure modeling and the study designs of health effect estimation.

Several limitations should be acknowledged in this study. First, there is potential measurement error because we used measured PM${}_{2.5}$ concentrations taken at a small number of fixed monitoring stations as a primary data source in both spatial exposure models. This concern was lessened in the multi-sourced exposure model by incorporating additional data, such as AOD, but the sparse monitoring stations operated in the study area likely contributed to the measurement errors. Furthermore, reliance on a sparse monitoring network might affect the observed mobility effects as well as the moderation of the mobility effect on personal exposure to PM${}_{2.5}$. Similarly, our findings might be subject to confounding factors, such as study participants’ age, gender and occupational status. The effect of stratification of exposure to different subpopulation groups needs to be further investigated. Third, we have stratified individuals’ long-term travel patterns into three groups based on both a spatial and temporal mobility metric, namely radius of gyration and time spent away from home. However, other mobility metrics, such as mean square displacement and entropy [73], could be used to quantify individuals’ long-term travel patterns, and its effect on the statistical analysis needs to be investigated. Lastly, our personal exposure assessment was limited to ambient PM${}_{2.5}$ concentrations for the purpose of illustration without the health effect assessments. In future work, we plan to include other pollutants that are known to have severe adverse health impacts, such as NO${}_{2}$ and O${}_{3}$, as well as the subsequent health outcomes, including flu symptoms.

## 5. Conclusions

This study suggests that there is a significant association between mobility and long-term exposure measurement error, although the mobility effect can be modified by individuals’ routine travel patterns and the spatiotemporal variability of air pollutants of concern. Our findings on the modification effect of mobility suggest that even accurately measured mobility may have little to no effect on exposure assessment for individuals with static travel patterns over a relatively homogeneous air pollution surface. Meanwhile, our findings underscore the significance of a mobility-based approach when a study involves individuals who regularly travel long distances and spend longer hours outside the home over spatially and temporally dynamic air pollutant concentrations. These findings suggest that investigators should pay greater attention to both individuals’ routine travel patterns and the spatial uncertainty of air pollution predictions to minimize long-term exposure measurement error.

## Figures and Tables

**Table 1:** Stratification of individuals’ travel patterns.

Travel Patterns	Static	Moderate	Active
	$r_{i}<R_{c},\tau_{i}<\Gamma_{c}$	$r_{i}\geq R_{c},\tau_{i}<\Gamma_{c}$	$r_{i}\geq R_{c},\tau_{i}\geq \Gamma_{c}$
		$r_{i}<R_{c},\tau_{i}\geq \Gamma_{c}$	

**Table 2:** The summary statistics of a total of 2013 study participants’ travel patterns quantified by the Radius of Gyration (RoG) and Non-home Time (NhT).

	Mean	SD	Min	Q1	Median	Q3	Max
RoG	6.27	3.60	0.08	3.76	5.51	8.13	24.34
NhT	5.84	2.91	0.03	3.34	6.37	7.94	16.21

SD denotes standard deviation; Q1 and Q3 denote the first and third quartile, respectively.

**Table 3:** The spatial mean and variability of PM${}_{2.5}$ concentration estimates from a single-sourced and multi-sourced exposure models.

Model		Mean	SD	Min	Q1	Median	Q3	Max
Single-Sourced	DM	7.15	3.11	1.25	4.86	6.71	8.77	16.58
	DSD	0.25	0.35	0.00	0.06	0.15	0.29	2.39
Multi-Sourced	DM	5.60	2.28	1.14	3.96	5.24	6.83	14.66
	DSD	1.16	0.58	0.20	0.68	1.11	1.48	3.13

DM denotes the daily mean and DSD denotes the daily standard deviation.

**Table 4:** Statistical models.

	Step 1	Step 2	Step 3
(Intercept)	7.23 ***	7.23 ***	7.21 ***
*Main Effects*	
Mobility-based Approach	0.03 **	−0.03	−0.00
Travel Patterns	−0.04 ***	−0.02	−0.00
Multi-sourced Exposure Model	−0.51 ***	−0.47 ***	−0.44 ***
*2-Way Interaction Terms*	
Mobility-based × Travel Patterns		0.03 *	0.00
Mobility-based × Multi-sourced		0.06 **	0.01
Travel Patterns × Multi-sourced		−0.07 ***	−0.10 ***
*3-Way Interaction Term*	
Mobility-based × Travel Patterns × Multi-sourced			0.05 *
AIC	10,702.71	10,672.50	10,674.81
BIC	10,744.66	10,735.42	10,744.73
Log Likelihood	−5345.35	−5327.25	−5327.41

*** *p* < 0.001; ** *p* < 0.01; * *p* < 0.05.

## Data Availability

The datasets (study participants’ mobility data) analyzed during the current study are not publicly available due to the privacy of individuals.
